# Case of Infanticide

**Published:** 1857-01

**Authors:** 


*Case of Infanticide.*—The public of the cities of Rochester and Buffalo, have been recently much excited by the arrest and examination of Dr. Augustus P. Biegler, of Rochester, (the crime taking place in Buffalo,) on a charge of murder.

Biegler is a well known homœopath in Rochester. Nearly four years since we alluded to his career in the pages of this Journal, as one infamous and criminal beyond measure, and declared our surprise that he should, even after conviction, receive the support of a respectable community. We trust he is now in a position to put a stop on future crimes.

The evidence given on the careful and prolonged examination before Coroner Nott, of this city, shows that Biegler was the seducer of a young girl named Emilie Murr; that in September last he brought her to Buffalo, where she took the name of Julia Rosendale, he assuming the name of Rosendale, also, on his visits to her. That on the night of the 19th of December, he took her from her boarding-house to a hotel on Exchange street, at about 10½, P. M. That at 12, midnight, the girl was very sick, though well when she entered the house; that her breathing was rapid and laborious, and she very much prostrated. Biegler then, or at 2, A. M., took her back to her boarding-house, and next morning left her under the medical charge of Dr. Warner, a well-known homœopath of this city. The girl lived until the evening of the 21st, when she gave birth to a dead child while herself *in articulo mortis.*

Wounds were described by a portion of the witnesses, both medical and laical, as existing upon the body of the child; and some witnesses also testified to a wound in the os uteri, and to inflammatory action in the bowels, though there were differences in the medical testimony on this point. There was also congestion of the right lung.

So far as we are able to form any opinion of this history of crime, we are inclined to give the following as the probable state of facts.

Biegler took the girl to the hotel and gave her chloroform. While she was under its influence, he attempted to rupture the membranes with a trocar, and probably inflicted the wounds found. From the shock of the operation, and (in all probability) from an unhappy effect of the chloroform, death resulted.

As we understand the law, the procuring the abortion was simply manslaughter. The death of the mother from wounds thus feloniously inflicted, constitutes murder, and it is probable that he will be indicted for that crime.

Biegler has once been in States Prison for arson, in setting fire to his own house.

